# Retraction: The impact of haulm killing on yield and quality of potato (*Solanum tuberosum* L.)

**DOI:** 10.1371/journal.pone.0274146

**Published:** 2022-09-14

**Authors:** The PLOS ONE Editors

The *PLOS ONE* Editors retract this article [1] because it was identified as one of a series of submissions for which we have concerns about authorship, competing interests, and peer review. We regret that the issues were not addressed prior to the article’s publication.

YBK did not agree with the retraction.
